# Outward depolarization of the microglia mitochondrial membrane potential following lipopolysaccharide exposure: a novel screening tool for microglia metabolomics

**DOI:** 10.3389/fncel.2024.1430448

**Published:** 2024-11-06

**Authors:** Kendra I. McGlothen, Rochelle M. Hines, Dustin J. Hines

**Affiliations:** Department of Psychology, Psychological and Brain Sciences & Interdisciplinary Neuroscience Programs, College of Liberal Arts, University of Nevada, Las Vegas, NV, United States

**Keywords:** microglia, mitochondrial membrane potential, lipopolysaccharide, emapunil, tetramethylrhodamine ethyl ester

## Abstract

Microglia are non-electrogenic immune cells that respond rapidly to protect the central nervous system (CNS) from infections, injuries, or other forms of damage. Microglia mitochondria are essential for providing the requisite energy resources for immune regulation. While fluctuations in energy metabolism are regulated by mitochondria and are reflected in the mitochondrial membrane potential (ΔΨm), there remains a lack of innovation in microglia-centric tools that capitalize on this. In this study, live imaging of microglia in acute slices from EGFP reporter mice expressing EGFP under the control of the fractalkine receptor (CX3CR1) promoter is combined with loading a fluorescent reporter of ΔΨm. Depolarizations in the ΔΨm were recorded after administering the well-characterized immune stimulant lipopolysaccharide (LPS). Microglia ΔΨm increased in distinctive phases with a relatively steep slope following LPS exposure. Conversely, the ΔΨm of neurons showed minimal regulation, highlighting a distinct microglia ΔΨm response to immune stimuli. Analysis of the depolarization of the microglia ΔΨm in the soma, branches, and endfeet revealed progressive changes in each subcellular domain originating in the soma and progressing outward. The inverse agonist emapunil attenuated the depolarization of the ΔΨm across states in a domain-specific manner. These findings emphasize the contribution of mitochondrial membrane dynamics in regulating microglial responses to immune stimuli. Further, this work advances a novel drug screening strategy for the therapeutic regulation of metabolic activity in inflammatory conditions of the brain.

## 1 Introduction

The emphasis in treating neurological diseases has predominantly centered on neurons, given their pivotal role in shaping behavioral responses (Huang et al., 2017; Guerram et al., 2016). However, increasing research has shown that glial cells contribute to behavioral outcomes under healthy and abnormal conditions (Hines and Haydon, 2013; McNeela et al., 2018; Peng et al., 2023). By comparison, non-electrogenic glial cells are known to perform many critical functions through active signaling pathways but have remained unexplored as therapeutic targets. This may be partly due to the limited available assays to measure essential glial signaling mechanisms and pathways in live tissue. Although previously regarded as passive support cells, much research has demonstrated that glial cells are highly dynamic and engage in extensive signaling (Khakh and Sofroniew, 2015; Barres, 2008; Fields et al., 2014). Microglia, as the resident immune cells of the brain, continuously surveil the tissue, rapidly modify their morphology in response to stimuli, engage in phagocytosis, and secrete cytokines, chemoattractants, and neurotrophic factors (Wake et al., 2009; Nimmerjahn et al., 2005; Kettenmann et al., 2011). The actions of microglia are necessary for normal brain development and homeostasis, and microglia perform several protective functions in nervous system damage and disease (Tremblay et al., 2011; Colonna and Butovsky, 2017). It is also recognized that prolonged or chronic microglia activation can contribute to disease pathogenesis (Block and Hong, 2005; Ransohoff, 2016), suggesting that interventions limiting microglia activation during chronic inflammation may provide therapeutic benefits.

Inflammation is an energetically expensive process, putting a strain on cellular energy production by mitochondria within immune cells like microglia (Mills et al., 2017). Toll-like receptor (TLR) ligands, such as lipopolysaccharide (LPS), promote activation of microglia and secretion of cytokines such as interleukin-6 (IL-6) and IL-1β to induce inflammatory responses, generate reactive oxygen species (ROS), and increase expression of factors such as major histocompatibility complex (MHC-I/II) and cluster of differentiation factors (CD80 & 86) (Turrin et al., 2001; Olson and Miller, 2004; Qin et al., 2005; Casals et al., 2007; Zhou et al., 2012). Recent studies in peripheral macrophages also indicate that mitochondria are necessary for initiating and maintaining immune responses beyond their role in generating ATP and metabolites (Seth et al., 2005; Breda et al., 2019). With relevance to inflammation, mitochondria also regulate cellular Ca^2+^ homeostasis, are the primary source of ROS, and orchestrate apoptosis, proliferation, and differentiation (Modesti et al., 2021; Banoth and Cassel, 2018; Wang et al., 2021). Under basal conditions, mitochondria maintain a relatively stable membrane potential through the function of proton pumps, and this transmembrane potential of protons is harnessed for the production of ATP (Zorova et al., 2018). Long-lasting drops in mitochondrial membrane potential (ΔΨm) signal bioenergetic stress and signal the release of factors that lead to apoptosis (Nicholls, 2004). ΔΨm can fluctuate up or down during normal physiological processes, providing a readout of cell function (Zorova et al., 2018).

Tetrameythlrhodamine ethyl ester (TMRE) (Crowley et al., 2016; Ehrenberg et al., 1988) is a cell-permeable dye that equilibrates across the membrane and accumulates into the mitochondrial matrix in an inversed proportion to ΔΨm. High concentrations of fluorochromes accumulate in depolarized mitochondria, and low concentrations accumulate in hyperpolarized mitochondria (Perry et al., 2011). TMRE is a potentiometric dye, and its fluorescence is directly proportional to mitochondrial membrane potential (ΔΨm), where higher TMRE fluorescence indicates a hyperpolarized state and lower fluorescence indicates depolarization. Based on the strong ties between mitochondrial function and immune responses, we hypothesized that microglia ΔΨm would provide a means to assay microglia signaling and function. To investigate the mitochondria dynamics of activated microglia, we prepared acute slices obtained from fractalkine reporter mice (Jung et al., 2000) and loaded them with the permeant dye TMRE. Microglia morphology and ΔΨm were imaged to establish a baseline before applying LPS. Following LPS exposure, we found that the microglia ΔΨm inclined with a steep slope angle at distinct periods. This increase in ΔΨm, reflected by elevated TMRE fluorescence, suggests an initial hyperpolarization and increase in mitochondrial activity as microglia respond to the immunological stimulus. In contrast, the ΔΨm of neighboring neurons showed minimal modulation, demonstrating a specific microglia ΔΨm response to immunological stimuli. The analysis of microglia ΔΨm depolarization in the soma, branches, and endfeet indicated gradual alterations in each subcellular domain, beginning in the soma and progressing outwardly to the endfeet. This progressive depolarization, marked by a decrease in TMRE fluorescence, may indicate mitochondrial dysfunction due to sustained stress or damage as the activation response continues. A ligand for the mitochondrial 18 kDa translocator protein (TSPO) attenuated the depolarization of the ΔΨm across states and in specified regions of the cell. These findings should be interpreted with the understanding that TMRE fluorescence dynamics reflect both the initial activation and potential subsequent dysfunction of mitochondria during microglial responses to stimuli.

TSPO is a protein located in the outer membrane of mitochondria and is predominantly expressed in the microglia of the brain—the expression of TSPO increases during inflammatory periods (Cosenza-Nashat et al., 2009; McNeela et al., 2018). There has been considerable discussion surrounding the various functions of TSPO in cellular physiology (Krueger and Papadopoulos, 1990; Bonsack and Sukumari-Ramesh, 2018; Tu et al., 2014). Nonetheless, evidence suggests that TSPO is involved in the permeability transition pore, neurosteroidogenesis, cell proliferation, and inflammation (Chelli et al., 2004; Kunduzova et al., 2004; Lacapère and Papadopoulos, 2003; Torres et al., 2000; Beinlich et al., 2000). Experimental studies using TSPO ligands like emapunil (XBD173) have demonstrated its protective effects in mouse models of neurodegeneration (Veiga et al., 2007; McNeela et al., 2018). Emapunil has been found to exert anxiolytic effects by enhancing GABAergic transmission through TSPO-regulated neurosteroid synthesis (Rupprecht et al., 2009). The association between TSPO and the mitochondrial permeability transition pore suggests that TSPO may contribute to regulating mitochondrial function (Sileikyte et al., 2011). The crucial role of mitochondria in initiating and sustaining immune responses is now recognized as a central mechanism in the development of inflammatory brain diseases (Angajala et al., 2018). The findings of this study will provide insight into the mitochondrial membrane dynamics that underlie microglia responses to immune stimuli. Examining how the ΔΨm in microglia is regulated during inflammation will provide insight into gliotic-specific screening techniques for neuroinflammatory diseases.

## 2 Materials and methods

### 2.1 Subjects

All experimental protocols were approved by the Institutional Animal Care and Use Committee (IACUC) of the University of Nevada Las Vegas. The University of Nevada Las Vegas vivarium is maintained at 70 degrees Fahrenheit, and the colony lights are on a 12-h light/dark cycle. Mice are provided access to food and water *ad libitum*. Slices were prepared from CX3CR1-EGFP transgenic mice aged 15–25 days postnatal. Pups were group housed with the dam until the time of experimentation. The CX3CR1-EGFP transgenic mice were sourced from Jackson Laboratory (Bar Harbor, ME, USA).

### 2.2 Slice preparation and solutions

Acute brain slices were prepared using a Vibratome and were cut at a thickness of 400 μm. Slices were equilibrated at room temperature (20–23.3°C) for 30 min before imaging in an oxygenated artificial cerebrospinal fluid (aCSF) containing (in mM): NaCl 126, KCl 2.5 or 4.2, NaHCO_3_ 26, glucose 10, MgCl_2_ 2, NaH_2_PO_4_ 1.25 and CaCl_2_ 2. To maintain consistency throughout the study, both baseline measurements and all treatments were performed using aCSF. TMRE was applied in aCSF, and although LPS was initially dissolved in saline, it was subsequently added to aCSF for bath application at a rate of 1–3 mL/min during acute slicing experiments. Slices were transferred to a recording chamber and perfused with oxygenated aCSF at a rate of 1–3 mL/min for maintenance.

### 2.3 Loading and treatment

The cell-permeant dye Tetramethylrhodamine ethyl ester (TMRE) was applied to acute slices at a concentration of 1 nM, prepared in 4 mL of artificial cerebral spinal fluid, for a duration of 5 min. LPS was dissolved in saline at a concentration of 10 μg/mL, and bath applied at a rate of 1–3 mL/min during acute slicing experiments. This concentration of LPS was selected based on its established ability to induce a robust inflammatory response in acute brain slices, as demonstrated in previous studies. The dose-dependent effects of LPS on different cell types have been studied across a range of concentrations from 100 ng/mL to 10 μg/mL to examine systemic inflammation and neuroinflammation (Qin et al., 2004; Lehnardt et al., 2002). Emapunil was dissolved in saline to a concentration of 50 μg/mL. In experiments where both LPS and emapunil were applied, the pretreatment of emapunil preceded LPS by 10 min. All reagents were obtained from Thermo Fisher Scientific (Waltham, MA, USA).

### 2.4 Live imaging

We performed imaging with a Nikon Eclipse e800 microscope fitted with a 40X-W/0.80 numerical aperture objective lens directly coupled to a Hamamatsu digital CMOS camera. EGFP was typically excited at 470 nM, and TMRE was excited at 552 nM. For acquiring images, the LED-driver LEDD1B did not exceed 1,000 mA. To address potential limitations of wide-field microscopy, measures were taken to minimize signal overlap and depth-dependent variations. Regions of interest were recorded in the neuronal cell body layer of hippocampal slices and compared to microglia, revealing significant differences in fluorescence signals. There was no photobleaching, nor was there any evidence of cellular damage while obtaining time-lapse images. The LED current limit was carefully monitored in all instances and kept comparable between all experiments. Imaging was conducted at a rate of 100 ms exposure, 200 ms delay, and a gain of 200.

### 2.5 Data and statistical analysis

ImageJ and Clampfit 10.7 were utilized to trace ROIs and measure the percent change from baseline in stack images. Statistical significance of differences in mean values were assessed by conducting a t-test, a repeated measures analysis of variance (ANOVA), or one-way ANOVA as appropriate. Differences between means were considered significant at values of *p ≤ 0.05, **p ≤ 0.01, ***p ≤ 0.001. Sample sizes were determined to ensure sufficient power to detect meaningful differences between groups, based on anticipated effect sizes and variability observed in preliminary experiments. This approach was taken to account for potential variability across samples, ensuring robust and reliable statistical outcomes.

## 3 Results

### 3.1 LPS exposure results in progressive increases in mitochondrial membrane potential in microglia but not adjacent neurons

**Figure 1:**
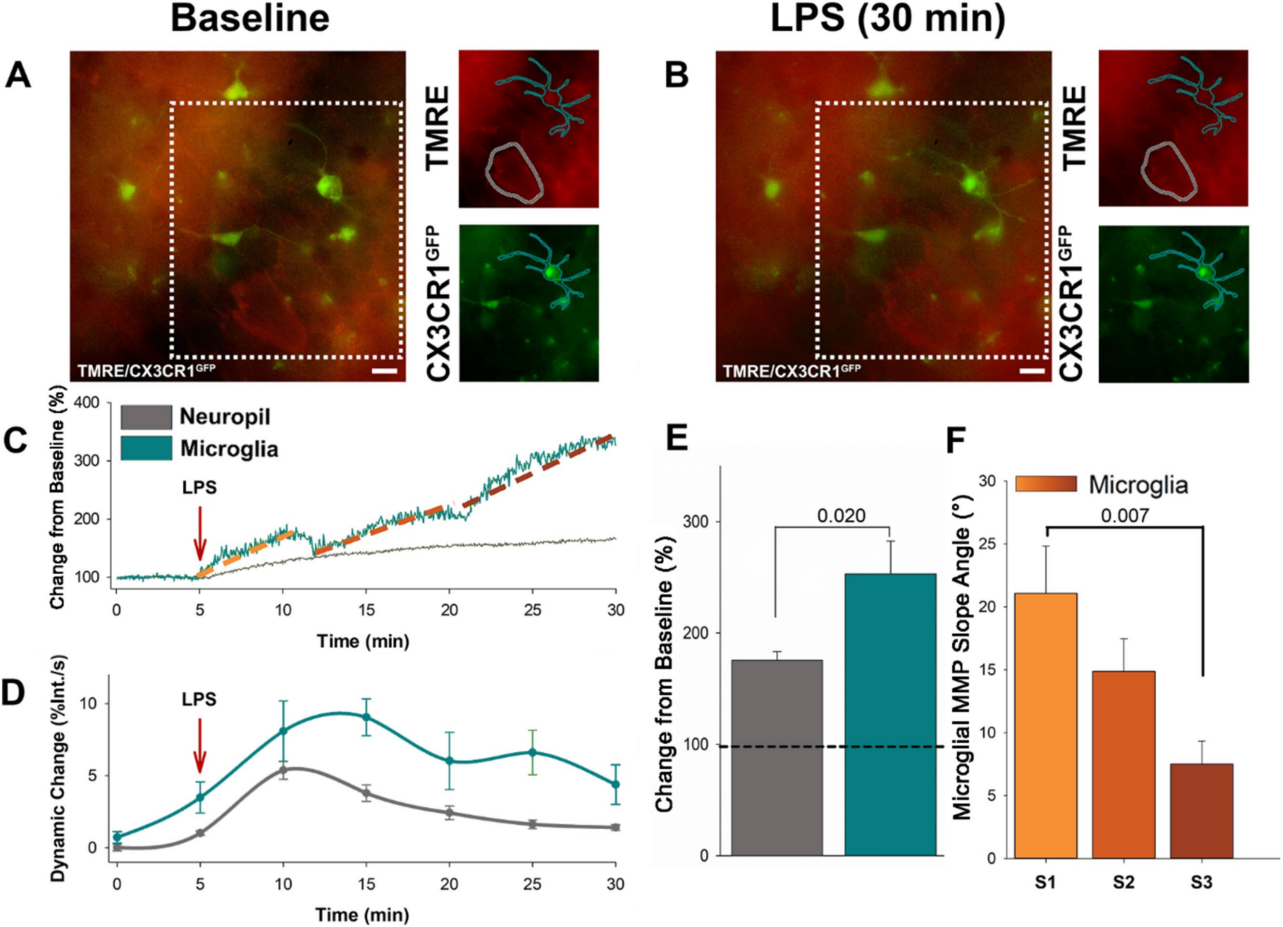
Microglia specific increases in ΔΨM occurs in progressive stages following LPS administration. **(A)** Representative baseline image showing microglia expressing EGFP, along with loading of TMRE in both microglia and adjacent neurons prior to LPS application. **(B)** Subsequent representative image of microglia and adjacent neurons following 30 min of LPS administration. **(C)** Following the application of LPS, the representative trace shows the ΔΨM in microglia dynamically increases in three progressive states (S1–3) over 30 min of exposure to LPS. By contrast the adjacent neurons showed only a slow and gradual rise. **(D)** Grouped data reveals dynamic change of the microglia ΔΨM surpasses adjacent neurons, exhibiting prolonged fluctuations compared to the initial change and subsequent decline in neurons (*p* < 0.002). **(E)** On average, microglia ΔΨM increases by close to 300%, while the adjacent neuronal ΔΨM increases by ~50% (*p* = 0.020). **(F)** The initial rise in microglia ΔΨM was the sharpest, and the slope angle decreased over the two following states (states begin at ~5, 13, 23 min following LPS administration). On average, the slope angle of the first state was significantly greater than the slope angle of the third state (*p* = 0.007).

To begin investigating the utility of using the ΔΨm to indicate the responsiveness of microglia to immune stimuli, we bath applied LPS to acute cortical slices prepared from CX3CR1-GFP mice loaded with tetramethylrhodamine ethyl ester (TMRE) to indicate changes in the ΔΨm. Timelapse recordings were taken over a duration of 5 min baseline followed by LPS (10 μg/mL) exposure for an additional 30 min (Figures 1A, B). We traced microglia cell profiles based on the GFP signal and examined changes in TMRE over the LPS time course compared to baseline. We found that LPS exposure resulted in a progressive increase in ΔΨm in microglia, with successive stages of escalating intensity (Figure 1C). On average, LPS exposure resulted in a 253.12% increase in intensity in microglia (Figure 1E). As a point of comparison, we also traced the profiles of surrounding neuronal somas and examined changes in TMRE over the LPS time course. In contrast to microglia, neurons showed a more gradual rise in ΔΨm (Figure 1C). LPS resulted in an average 50% increase in TMRE intensity in neuronal somas (Figure 1C). Microglia underwent a significant increase in ΔΨm following LPS exposure compared to ΔΨm in adjacent neurons (*p* = 0.020, *F* = 6.526, *n* = 10; Figure 1E). Microglia exhibited prolonged fluctuations in ΔΨm, while adjacent neurons showed only an initial change followed by a steady decline (Figure 1D). To calculate the dynamic change in ΔΨm, the percent change in TMRE intensity was measured over time and expressed as a rate of change (percent change per second) throughout the time course. This transformation allowed for the quantification of the relative fluctuations in ΔΨm and provided a comparison between microglial and neuronal responses. The microglia ΔΨM progressively increased in distinctive states, characterized by a steep rise in ΔΨm and separated by a brief plateau. The first state (S1) begins within 6.06 min of LPS application, with an average duration of 2.02 min. The second state (S2) starts within 14.06 min of LPS application, with an average duration of 2.07 min. The third state (S3) begins within 23.50 min of LPS application, with an average duration of 3 min. We found that the initial state was characterized by the steepest rise in ΔΨm, with a significantly greater ΔΨm slope angle compared to state 3 (*p* = 0.007, *F* = 5.726, *n* = 10; Figure 1F). The increase in the intensity of TMRE in microglia following LPS suggests more metabolic demand within microglia compared to the surrounding neuronal population. The progressive states in microglia ΔΨm indicate that these cells are responding to the immune stimulus in a regulated fashion.

### 3.2 Depolarization of the microglia ΔΨm progresses outward from the soma following LPS exposure

**Figure 2:**
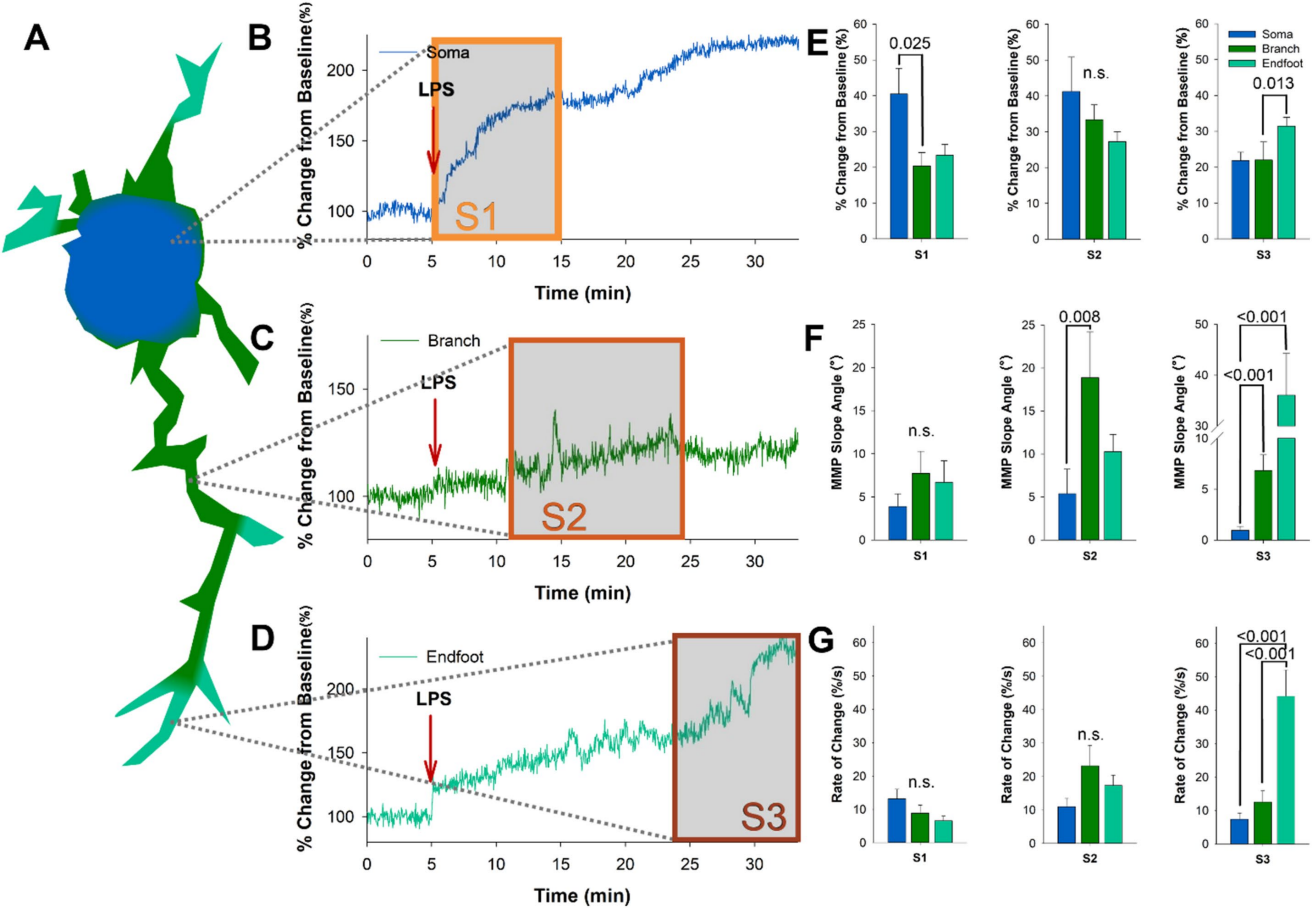
The progressive states observed following LPS administration represent radiating depolarization of the microglia ΔΨM, beginning in the soma and progressing to the endfeet. **(A)** Schematic of the subcellular progression of microglia ΔΨM changes in regions of interest following LPS administration. **(B)** The intensity of the ΔΨM in the microglia soma climbed rapidly between 5 and 15 min post LPS administration, which temporally matches S1. **(C)** The sharpest rise in microglia branch ΔΨM occurs after 11 min of LPS administration. **(D)** The intensity of the ΔΨM in the microglia endfeet climbed rapidly between 24 and 30 min post LPS administration, which temporally matches S3. **(E)** Examination of average percent change from baseline in each subcellular domain during the three progressive stages. During S1 we see the largest magnitude change in the soma (*p* = 0.025), while during S3 the largest magnitude change is in the endfeet (*p* = 0.013). **(F)** Quantification of the maximum ΔΨM slope angle shows that microglia branches have the sharpest rise during S2 (*p* = 0.008), and both branches and endfeet have significantly greater slope angles during S3 when compared to the soma (*p* < 0.001; *p* < 0.001). **(G)** Examination of the rate of change in the microglia ΔΨM also supports a significant difference in the microglia endfeet during S3 (*p* < 0.001).

Dynamic depolarizations were recorded in the ΔΨm of microglia following LPS application, characterized by temporally separated progressive states (Mean 365.5, 844, and 1,410 s). To examine the subcellular regulation of microglia in response to LPS, we examined ΔΨM in the soma, branches, and endfeet of microglia (Figure 2A) in acutely prepared slices from CX3CR1-GFP mice loaded with TMRE. When reviewing the LPS-induced changes in ΔΨM in microglia somas, we found that the sharpest rise occurred early following application, within 6.06 min, which was followed by a more gradual rise throughout the remainder of the 30-min time course (Figure 2B). Analysis of ΔΨM in microglia branches following LPS application showed a delayed increase, commonly characterized by a sharp rise between 13.03 and 15.10 min (Figure 2C). Microglia endfeet showed the most delayed change in ΔΨM following LPS, with the sharpest rise occurring between 22 and 25 min (Figure 2D). Next, we examined the average percent change related to our identified progressive states within each subcellular domain. We found that the microglia soma undergoes a significant shift in ΔΨM during state 1 following LPS, while the microglia endfeet undergo a substantial shift in the ΔΨM during state 3 (S1 *p* = 0.025, *F* = 4.714, *n* = 10; S3 *p* = 0.013, *F* = 8.743, *n* = 10; Figure 2E). Analysis of the maximum ΔΨM slope angle of each subcellular domain in each state revealed that the branches undergo the steepest slope changes during state 2 and state 3 (S2 *p* = 0.008, *F* = 9.757, *n* = 15; S3 *p* < 0.001, *F* = 23.321, *n* = 15; Figure 2F). In contrast, microglia endfeet undergo the steepest slope change during state 3 (S3 *p* < 0.001; Figure 2F). We also examined the rate of change ΔΨM in each microglia subcellular domain with respect to the progressive states and found that the endfeet show the most significant rate of change during state 3 (S3 *p* < 0.001, *F* = 23.321, *n* = 10; Figure 2G). These data reflect a progressive depolarization of the ΔΨM in microglia following exposure to an immune stimulant, which can be separated temporally into distinct states and spatially into subcellular domains. Changes in microglia ΔΨM occur first in the soma, then radiate outward through the branches and finally to the endfeet over 30 min following LPS application.

### 3.3 Emapunil attenuates the progressive increase in microglia ΔΨm following LPS exposure

**Figure 3:**
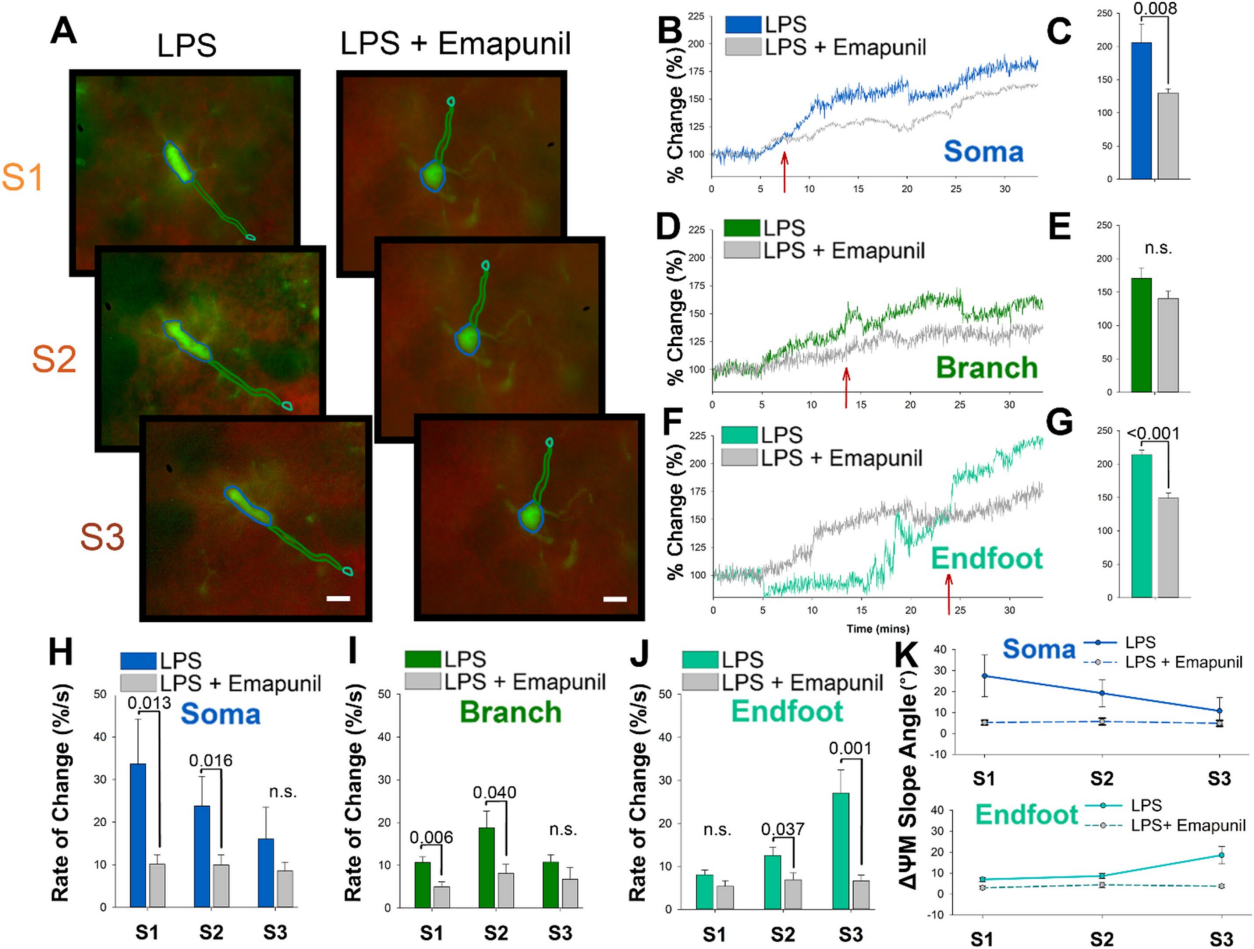
The TSPO ligand emapunil shunts the somatic to endfoot progression ΔΨM depolarization in microglia endfeet. **(A)** Representative timelapse images of slices treated with LPS (left) or LPS plus emapunil (right) during states 1–3. **(B)** In the microglia soma, emapunil attenuated the percent change from baseline of the ΔΨM caused by LPS within 7 min of administration. **(C)** On average, treatment with emapunil resulted in a significantly smaller change in the soma ΔΨM caused by LPS compared with LPS alone (*p* = 0.008). **(D,E)** Emapunil did not significantly impact the change in branch ΔΨM caused by LPS. **(F)** Emapunil also influences the change in ΔΨM caused by LPS in the endfeet. **(G)** On average, emapunil significantly decreases the percent change in ΔΨM in microglia endfeet following LPS administration (*p* < 0.001). **(H)** Quantification of the rate of change comparing LPS and LPS plus emapunil treated slices in the microglia soma (S1 *p* = 0.013; S2 *p* = 0.016). **(I)** Quantification of the rate of change comparing LPS and LPS plus emapunil treated slices in the microglia branches (S1 *p* = 0.006; S2 *p* = 0.040). **(J)** Quantification of the rate of change comparing LPS and LPS plus emapunil treated slices in the microglia endfeet (S2 *p* = 0.037; S3 *p* = 0.001). **(K)** The slope angle of the microglia ΔΨM was significantly decreased by emapunil in the microglia soma during states 1 and 2, and the microglia endfeet during state 3 (S1 *p* = 0.007; S2 *p* = 0.001; S3 *p* < 0.001).

We next wanted to determine if the depolarization of microglia ΔΨm in response to LPS could be modulated. The outer mitochondrial membrane protein TSPO is known to signal inflammatory transcriptional pathways and interact with reactive oxygen species (ROS) as a vital part of the microglia inflammatory response (Cosenza-Nashat et al., 2009). We examined the effects of the well-characterized TSPO inverse agonist, emapunil, which has previously been demonstrated to exert neuroprotective effects (Gong et al., 2019; Leva et al., 2017). Emapunil is classified as an inverse agonist due to its ability to reduce the constitutive activity of TSPO receptors, thereby decreasing baseline TSPO activity. This is particularly relevant, as the modulation of TSPO activity by emapunil could directly influence the inflammatory response in microglia. Application of emapunil showed no significant impact on baseline ΔΨM in either microglia or neurons (Supplementary Figure 1). By suppressing the intrinsic activity of TSPO, even in the absence of a ligand, emapunil may effectively mitigate the mitochondrial dysfunction and depolarization typically observed in microglia following LPS exposure. For these studies, acutely prepared slices from CX3CR1-GFP mice were loaded with TMRE and pretreated with emapunil before LPS exposure. Timelapse imaging of microglia pretreated with emapunil showed a visible decrease in the intensity of TMRE following LPS exposure compared with LPS alone (Figure 3A). The change in intensity of the ΔΨM in the microglia soma is substantially attenuated following LPS when slices were pretreated with emapunil (Figure 3B). The state 1 rise in ΔΨm of the microglia soma is notably attenuated by 7 min following LPS exposure (red arrow Figure 3B). On average the percent change in ΔΨm in the microglia soma following LPS were significantly attenuated by emapunil pretreatment (*p* = 0.008, *F* = 2.634, *n* = 10; Figure 3C). Emapunil did not significantly affect the ΔΨm change in microglia branches following LPS (Figures 3B-E), but it significantly influenced the ΔΨm change in the endfeet (*p* < 0.001, *F* = 6.273, *n* = 10; Figures 3F-G). Quantification of the rate of change in the microglia soma and branches showed significant attenuation during S1 and S2 following emapunil treatment (S1 *p* = 0.013, *n* = 10; S2 *p* = 0.016, *n* = 10; Figures 3H-I), and in the endfeet during S2 and S3 (S2 *p* = 0.037, *n* = 10; S3 *p* = 0.001, *n* = 10; Figure 3J). Additionally, emapunil significantly decreased the slope angle of ΔΨm in the microglia soma during states 1 and 2, and the microglia endfeet during state 3 (S1 *p* = 0.007; S2 *p* = 0.001; S3 *p* < 0.001, *n* = 10; Figure 3K).

## 4 Discussion

This study investigates the mitochondrial dynamics of activated microglia to examine if the microglia ΔΨM can be used as an assay to indicate microglia signaling and function. We found that microglia-specific increases in the ΔΨM occurred in progressive states following LPS administration. The initial (S1), intermediate (S2), and late (S3) microglia ΔΨM states reflect a radiating depolarization that begins in the soma and progresses to the endfeet. Previous research has indicated that LPS exerts dose-dependent effects on various outputs, influencing cytokine production, microglia activation, and inflammatory responses (Smith et al., 2016; Cho et al., 2016). In our study, we rigorously controlled LPS toxicity by screening multiple doses, and our findings remained consistent with a specific LPS dose (10 μg/mL), ensuring reliability in our experimental conditions. The application of a TSPO inverse agonist, emapunil, shunts the somatic to endfoot progression of the ΔΨM. A significant difference in the microglia ΔΨM is eminent in the transition between states and indicates a divergence in metabolic activity during the initial (S1) and late states (S3) of microglia activation.

Recent advancements in our understanding of neuroimmunology have departed from the traditional dualistic classifications of microglia activation (Paolicelli et al., 2022). In the past, microglia activation was categorized as either resting or activated, failing to account for the intricate and multifaceted nature of microglia responses. Microglia have context-dependent differential states (Tay et al., 2017); however, morphology alone cannot determine the state of activation. This study demonstrates that microglia metabolic states based on mitochondria can be quantified. Further, the divergence in the ΔΨM highlights the temporal dynamics of microglia activation, revealing the nuanced metabolic changes that occur during different phases.

Prior studies have implicated mitochondrial dynamics in the activation of the microglia system in various neuroinflammatory diseases (Culmsee et al., 2019; Park et al., 2013) yet isolating the most effective intervention point during microglia activation remains a significant challenge. While previous research has mainly focused on the mitochondrial dynamics of neurons (Su et al., 2010; Prehn, 1998; Ullah et al., 2012; Saeed et al., 2008), our findings indicate that changes in the ΔΨM of neurons plateau 9–16 min following LPS administration, making microglia a prime target for driving late-stage metabolic activity. This is supported by the dynamic depolarizations we observed in microglia ΔΨM at three time points following LPS administration, indicating a recurrent change in mitochondrial metabolism. These insights provide valuable information about microglia states during disease and highlight potential therapeutic points of intervention.

Maintaining mitochondrial health involves several dynamic behaviors, including fusion, fission, transport, and mitophagy. Initially, these processes appear mechanistically distinct from the biochemical and metabolic processes occurring within the organelle. However, previous research has shown a strong link between mitochondrial metabolism and dynamics (Mishra and Chan, 2016; West et al., 2011; Banoth and Cassel, 2018; Breda et al., 2019). Mitochondrial dynamics play a role in metabolic regulation, as mitochondria can undergo morphological change during periods of high energy demand and create more extensive interconnected networks to enhance ATP production. Changes in mitochondrial morphology support the trafficking of mitochondria to synapses in neurons, thereby accommodating the energy demands of neurons (Sheng and Cai, 2012). Interestingly, interaction sites between neuronal cell bodies and microglia processes have been identified as specialized nanoarchitectures that engage in purinergic signaling (Cserép et al., 2020). The dynamic mobility of microglia processes toward inflammation sites (Hines et al., 2013) led us to hypothesize that the ΔΨM could indicate shifts in energy metabolism throughout microglia processes.

This necessitates an exploration of the metabolic dynamics in various subcellular regions. We investigated region-specific depolarizations of microglia ΔΨM at the soma, branches, and endfeet. The results showed substantial increases in the late mitochondrial state (S3) of microglia cells, progressing outward from the soma to the endfeet following LPS administration. This aligns with prior research indicating increased mitochondrial fragmentation following chronic LPS exposure (Nair et al., 2019), suggesting alterations in mitochondrial formation. This begs the question of whether the ΔΨM in microglia could signify these dynamic shifts in microglia behavior.

Our data revealed a significant increase in the intensity of the ΔΨM in the microglia soma during state 1, followed by a plateau in ΔΨM intensity across consecutive states. This finding suggests a reduction in the metabolic activity of the microglia soma following the initial activation. We furthermore observed state-dependent variations in ΔΨM among different subcellular regions within microglia. During state 2, a significant difference in the slope angle and rate of change of the microglia ΔΨM was recorded in the branches compared to the soma, signifying a rapid and dramatic change in the microglia branches following the onset of activation. The late mitochondrial state also revealed notable differences in the intensity, slope angle, and rate of change in the microglia ΔΨM following LPS administration. These findings suggest dynamic metabolic activity shifts from the microglia soma to the endfeet during microglia activation.

We observed an increased depolarization of the ΔΨM at microglia endfeet, providing insights into mitochondrial translocation during microglia activation. Additionally, brief yet significant increases in the slope angle of the ΔΨM in microglia branches indicated a spatial hotspot of mitochondrial activity during state 2. This finding contrasts the prolonged recordings in early and late microglia ΔΨM states, which showed a consistent shift in the ΔΨM slope angle that remained increased throughout the imaging session. Changes in the duration of mitochondrial activity among microglia subcellular regions suggest a relocation of mitochondria from the soma to the endfeet during neuroinflammation. Considering the highly active profiles of both mitochondria and microglia, it is conceivable that as microglia become activated and undergo morphological changes, mitochondria engage in dynamic behaviors that alter the spatial location of microglia mitochondria and, consequently, the microglia ΔΨM.

TSPO has been suggested to influence the movement of mitochondria by interacting with ligands to regulate several molecular biological processes, including the generation of mitochondrial reactive oxygen species (ROS), ΔΨM, and ATP production (Papadopoulos et al., 2006; Rupprecht et al., 2009; Cosenza-Nashat et al., 2009). Our research investigated how changes in energy metabolism during microglia response to LPS can be modified by an inverse agonist for TSPO. Previous studies have shown that using TSPO ligands can reduce neuroinflammation after LPS administration (Azrad et al., 2019), making emapunil appropriate for our study. We found that emapunil administration attenuated the outward depolarization of the microglia ΔΨM at every time point. Additionally, emapunil led to a significant decrease in the rate of change of ΔΨM during the initial (state 1) and intermediate (state 2) mitochondrial states in both the microglia soma and branches, suggesting that emapunil restored metabolic activity closer to a baseline physiological state.

Similarly, the recordings of microglia endfeet following treatment with emapunil exhibited a decrease in the rate of change of microglia ΔΨM during states 2 and 3. This shift contrasts with the pattern seen in LPS-administered groups, where endfoot mitochondrial activity increased and somatic mitochondrial activity decreased.

The observed fluctuations in microglia ΔΨM suggest complex regulatory mechanisms, potentially involving emapunil’s modulation of the NF-κB signaling cascade to dampen proinflammatory cytokine synthesis and secretion. While alterations in the slope angle of ΔΨM in different microglial subcellular regions provide valuable insights, further correlation with other functional readouts, such as cytokine release or phagocytic activity, could enhance our understanding of microglial response dynamics to emapunil. The morphological changes observed in the LPS-administered groups, characterized by cell body enlargement and process retraction (Chen et al., 2012; Kloss et al., 2001), contrast with the more ramified morphology seen in the LPS and emapunil group. This suggests that emapunil may indeed mitigate the morphological alterations induced by LPS, potentially preserving microglial homeostasis. However, further analysis is warranted to comprehensively assess the extent of these morphological changes and their functional implications in response to emapunil treatment. While the administration of emapunil resulted in a decrease in microglia ΔΨM, the representative traces still exhibited dynamic fluctuations. The sustained late-state activity of the microglia ΔΨM, even in the presence of emapunil alongside LPS, suggests that emapunil is not inducing significant cellular toxicity. This continued metabolic activity indicates that the observed effects on ΔΨM are more likely due to the therapeutic action of emapunil rather than cellular death. These findings suggest that this novel approach to examining microglia mitochondrial activity provides a reliable output that could be used as a screening technique for potential therapeutic drugs. This experimental paradigm elucidates microglia mitochondrial activity’s spatial and temporal dynamics before, during, and after microglia activation. However, further investigation is needed to understand how other commonly reported neuroprotective drugs impact the metabolic activity of activated microglia during the onset of neuroinflammation.

### 4.1 Limitations and future directions

While the current study offers a novel approach with valuable insights, it also has several limitations. Firstly, although the ΔΨM can provide information regarding a cell’s mitochondrial function and energy production, it only offers a partial view of the overall cellular metabolic state. Cellular metabolism is a multifaceted process influenced by various factors that work in tandem to reach a metabolic equilibrium. In this intricate balance of biochemical reactions, the ΔΨM represents a critical checkpoint closely tied to energy production through oxidative phosphorylation. Future studies could benefit from integrating complementary assays such as ATP level measurements, ROS production, mitochondrial respiration rates, and glycolytic flux. These approaches would offer a more comprehensive understanding of cellular metabolism, providing additional context to the changes observed in ΔΨM during microglial activation. Secondly, this study investigates the microglia ΔΨM under acute inflammatory conditions. While acute models provide valuable insights into the immediate microglial responses, they may not fully capture the prolonged and complex metabolic changes associated with chronic neuroinflammation. Future research should incorporate chronic inflammatory models to better capture the sustained metabolic and functional alterations in microglia that occur during prolonged neuroinflammatory conditions, providing a more complete understanding of these complex processes. While we did not use explicit neuronal markers, our methodology involved recording from the neuronal cell body layer of hippocampal slices, providing a comparative analysis with microglia. The observed differences in TMRE fluorescence between neurons and microglia support the validity of our findings. To gain further insight additional investigation using mito::mKate2, a red fluorescent protein that can mark mitochondria inside cells, has the potential to offer valuable insights into the dynamic behavior of microglia during chronic inflammatory conditions. This research could contribute to a more comprehensive understanding of how microglia metabolic states evolve and resolve over time. Additionally, while this study focused on ΔΨM changes, future research should consider integrating other functional readouts such as cytokine release and phagocytic activity. These complementary assays could provide a more holistic view of microglial activity and its broader physiological relevance under inflammatory conditions. Lastly, while the technique employed in this study sheds light on mitochondrial dynamics during the early phases of microglia activation, it is crucial to emphasize that microglia do not operate in isolation. The role of other glial cells, such as astrocytes, in maintaining the brain’s metabolic environment during the microglia mitochondrial states discussed here warrants further exploration. The interactions and interplay between different glial cell types are likely integral to comprehending the complex metabolic responses in the context of neuroinflammation.

In summary, our study’s findings provide a novel glimpse into the intricate dynamics of microglia responses during the onset of neuroinflammation. The observed temporal and spatial changes in microglia ΔΨM underscore the remarkable adaptability of these immune cells in the face of neurological challenges. Specifically, the translocation of the dynamic properties of the ΔΨM from the soma to the endfeet during inflammation suggests a finely tuned metabolic response to support immune activities efficiently. Such insights into the spatial redistribution of energy resources within microglia can potentially revolutionize our understanding of neuroinflammatory diseases. Furthermore, the current lack of effective treatments for neuroinflammatory conditions emphasizes the urgency of our research. Our results open a viable path for creating brand-new treatment approaches by illuminating the complex metabolic states and actions of microglia during neuroinflammation. Targeting certain microglia states, like those seen in our work, might lead to more efficient therapies for various neurological conditions. As a result, our research advances our understanding of microglia biology and directly affects clinical investigation for more effective therapies for neuroinflammatory disorders. In addition to its therapeutic potential, our novel approach, centered on monitoring microglia mitochondria activity, provides a valuable tool for drug screening. The ability to assess the impact of potential neuroprotective drugs on microglia ΔΨM and the spatial distribution of metabolic activity offers a reliable and relevant screening technique. This novel approach can accelerate the advancement of therapies that regulate microglia responses in diverse neurological disorders. In conclusion, the contributions of our study enhance our comprehension of microglia biology, present potential pathways for more efficient therapeutic interventions, and establish a beneficial framework for future attempts in drug discovery pertaining to neuroinflammatory disorders.

## Data Availability

Data is stored at the National Supercomputing Center at UNLV and data will be released upon request by any qualified and interested researcher, at the discretion of the PI.

## Supplementary material

SUPPLEMENTARY FIGURE 1 Emapunil application shows no significant impact on baseline ΔΨM in microglia and neurons. **(A)** Representative baseline image showing microglia expressing EGFP in the neuronal cell body layer of the hippocampus. Inset images outline regions of interest for microglia and adjacent neurons. **(B)** Following the application of emapunil, the representative trace shows minimal change in ΔΨM from baseline in both microglia (blue) and neurons (gray). **(C)** Grouped data graph demonstrates no significant difference between microglia and neuronal ΔΨM following emapunil application compared to baseline activity, indicating emapunil does not significantly alter mitochondrial polarization in either cell type.
